# Selective retina therapy for central serous chorioretinopathy with focal choroidal excavation: a retrospective case series of six eyes

**DOI:** 10.1186/s40942-025-00741-x

**Published:** 2025-11-21

**Authors:** Seung Hee Jeon, Young-Jung Roh

**Affiliations:** Department of Ophthalmology and Visual Science, Yeouido St. Mary’s Hospital, College of Medicine, The Catholic University of Korea, 10, 63-ro, Yeongdeungpo-gu, Seoul, 07345 Republic of Korea

**Keywords:** Central serous chorioretinopathy, Focal choroidal excavation, Selective retina therapy

## Abstract

**Background:**

We evaluated the clinical outcomes of selective retina therapy (SRT) in patients with central serous chorioretinopathy (CSC) who had nonconforming focal choroidal excavations (FCEs).

**Methods:**

Among the 204 patients with chronic CSC who underwent SRT between June 2023 and February 2025, six patients (2.9%) with nonconforming FCEs were analyzed. Using a Q-switched Nd: YLF 527-nm laser device, SRT was applied at leaking points or in leakage areas according to the features observed on fundus fluorescein angiography. The mean best-corrected visual acuity (BCVA), central foveal thickness (CFT), subfoveal choroidal thickness height, and mean deviation (MD) of retinal sensitivity were measured during the follow-up period.

**Results:**

Among the six patients, five patients showed complete subretinal fluid (SRF) resolution, while one patient demonstrated minimal persistent SRF during the follow-up period. Four patients showed an improvement in BCVA, while BCVA remained unchanged in the other two patients. All patients showed improvement in the MD of retinal sensitivity during the follow-up period. Among the six patients, two patients showed complete SRF resolution after a single session of SRT, while the other four patients required multiple SRT sessions. No SRT-related adverse events were observed during the follow-up period.

**Conclusion:**

SRT produced favorable outcomes in the treatment of patients with CSC and nonconforming FCEs.

## Introduction

Central serous chorioretinopathy (CSC) is a retinal disorder marked by the idiopathic accumulation of subretinal fluid (SRF), which results in serous detachment of the neurosensory retina. This condition may be accompanied by pigment epithelial detachment (PED), although it can also occur independently, with or without SRF [1]. Epidemiological data have indicated a strong male predominance, with an annual incidence rate of 9.9 per 100,000 in men, which is approximately six times higher than the rate of 1.7 per 100,000 that is observed in women [2]. Various factors have been implicated as contributing to the development of CSC, including systemic corticosteroid use, psychological stress, Type-A personality traits, and a hereditary predisposition [3, 4].

Focal choroidal excavation (FCE) was first reported as a localized area of excavation in the choroidal layer [5]. Previous reports have shown that FCE is associated with various macular diseases, such as CSC and choroidal neovascularization (CNV) [6, 7]. Although the prevalence of FCE in patients with CSC is not clear, Suzuki et al. reported that 7 of 248 eyes (2.8%) with CSC was identified as having an FCE [8]. Considering that the incidence of CSC is very low, the CSC with FCE is expected to be very rare. An FCE is defined as an area of choroidal depression that is observed along the retinal pigment epithelium (RPE)/Bruch’s membrane complex on optical coherence tomography (OCT), in the absence of any history of trauma, infection, or inflammation. Based on OCT findings, FCE is categorized as *conforming* when photoreceptor tips contact the RPE surface, and as *nonconforming* when a gap is present between the photoreceptor tips and the RPE [7].

In terms of the pathogenesis of CSC, extensive choroidal hyperpermeability and RPE dysfunction is known to be causes of SRF and PED [1]. Acute CSC frequently resolves spontaneously, with good visual recovery occurring within 1–4 months even without treatment [9]. However, chronic CSC results in persistent SRF, which can cause irreversible vision loss due to diffuse RPE atrophy and subretinal fibrosis [10]. Therefore, to prevent further visual impairment, SRF elimination is crucial.

Clinically, several CSC treatment options have been used, including photodynamic therapy (PDT), conventional laser photocoagulation, subthreshold lasers, selective retinal therapy (SRT), and intravitreal anti-vascular endothelial growth factor (anti-VEGF) [11]. Conventional laser treatment is useful to remove SRF, but carries the risk of causing permanent damage to the retina and choroid, which can lead to central or paracentral scotoma and the development of CNV [12, 13]. Although PDT has shown good clinical outcomes in many studies [14, 15], adverse effects, such as acute vision loss due to RPE atrophy or choroidal ischemia, have been reported after both full-dose and reduced-dose PDT [16].

To avoid photoreceptor damage from conventional laser photocoagulation, SRT delivering a 527-nm wavelength laser with a pulse duration of 1.7 µs has been developed. SRT induces selective RPE damage, which induces restoration by development of a new RPE layer, while sparing the photoreceptors. Since RPE dysfunction is known to cause CSC, the development of a new RPE layer in the SRT-treated area might play a role in resolving SRF by forming a new outer blood–retinal barrier [17, 18]. To date, SRT is the only treatment that has been approved by the Korean Ministry of Food and Drug Safety for the treatment of CSC.

Although many papers have been published on the treatment of CSC, therapeutic reports specifically addressing CSC with FCE are very rare. Since SRT has shown favorable clinical outcomes in patients with CSC in previous reports [11, 17, 19, 20], the present study aimed to evaluate the clinical outcomes of SRT in patients with CSC and nonconforming FCEs.

## Materials and methods

We retrospectively reviewed the medical notes of 204 patients with CSC who underwent SRT between June 2023 and February 2025. All patients had experienced symptoms for ≥ 3 months and showed SRF in the central macula. All patients provided written informed consent for the SRT procedure as part of routine clinical care. The study was conducted in accordance with the Declaration of Helsinki and approved by the Institutional Review Board of Yeouido St. Mary’s Hospital, The Catholic University of Korea (approval number: SC25RISI0077). Given the retrospective design, the requirement for additional informed consent for data use was waived.

We selected patients who had undergone SRT and who met al.l of the following inclusion criteria: eyes with (1) visual symptoms for > 3 months; (2) the presence of nonconforming FCE observed on OCT; (3) focal or diffuse hyperfluorescent leakage observed on fundus fluorescein angiography (FFA) or indocyanine green angiography (ICGA); (4) availability of medical records for > 3 months of follow-up after SRT. We excluded (1) eyes with coexisting macular diseases, including age-related macular degeneration, polypoidal choroidal vasculopathy, or any evidence suggestive of CNV, such as retinal exudates, hemorrhages, or the presence of a double-layer sign on OCT (2), eyes that had previously undergone treatment with conventional laser or PDT; (3) eyes treated with intravitreal anti-VEGF injection within 12 weeks before SRT; (4) eyes of patients with local or systemic steroid use within 1 year prior to SRT.

Clinical data in the medical records, including best-corrected visual acuity (BCVA) swept-source OCT (DRI OCT Triton, Topcon, Tokyo, Japan), color fundus photography (CFP) (CF-60UVi; Canon Inc., Ota, Japan), fundus autofluorescence (FAF), FFA, ICGA (HRA2; Heidelberg Engineering, Dosenheim, Germany), compass microperimetry (CMP; Centervue, Padova, Italy) were reviewed. To assess the response to SRT, all patients underwent slit-lamp examination and Snellen BCVA testing, CFP, FAF, and OCT at baseline and at each visit. OCT was used to detect the presence of SRF, to identify FCEs, and to measure the central foveal thickness (CFT), subfoveal choroidal thickness (SFCT), as well as the excavation depth and width of the FCE by using a 7 × 7-mm² volume scan. SFCT was defined as the distance from the basal boundary of the RPE–Bruch’s membrane complex to the choroid–scleral junction beneath the fovea. When the FCE involved the foveal center, SFCT was measured at the nearest uninvolved location adjacent to the fovea. FFA and ICGA were used to verify leakage sites and to establish the diagnosis of CSC. Additional FFA was used to titrate the pulse energy by observing the angiographic features of test spots. Furthermore, microperimetry examination was performed when the SRF had resolved and every 6 months during the follow-up period. Moreover, 10 − 2 CMP examination was used to show a full threshold on the central 10° visual field with infrared imaging, using an automated perimeter with active retinal tracking. The mean deviation (MD) of retinal sensitivity was automatically calculated in CMP. Microperimetry testing was considered reliable when the false-positive rate did not exceed 18% [21].

A single physician (YJR) performed SRT using a Q-switched Nd: YLF 527-nm laser device (Macufocus; Threshold, Seoul, Republic of Korea), with a spot-size diameter of 200 μm, single micropulse duration of 1.7 µs, and a pulse-repetition rate of 100 Hz. This laser device has been approved for the treatment of CSC by the Korean Ministry of Food and Drug Safety. A retinal contact lens (Ocular Mainster Focal/Grid; Ocular Instruments, Bellevue, WA, USA) was used.

Individual differences in laser energy absorption due to differences in retinal pigmentation can lead to differences in the threshold at which RPE damage occurs across patients. Two clinical endpoints, including an “invisible spot on fundoscopy” and a “visible spot on FFA” have been used as a titration protocol to identify adequate SRT spots that will selectively damage the RPE while sparing the neurosensory retina [17]. We preformed SRT by adjusting the number of micropulses as well as the power of pulse energy by using a fundus image-guided strategy, as previously described [22]. Briefly, preliminary test spots with 20-µJ increments of pulse energy over a fixed number of micropulses, either three or 10, in pairs of the same pulse energy, were created around the arcade vessels. Adequate SRT spots were ophthalmoscopically invisible during test spot irradiation; some “barely visible” spots were observed on CFP by about 1 h after irradiation. The lowest pulse energy of the 10-micropulse bundle, where the test spot was barely visible, was chosen as the power setting for the treatment spot, while the same pulse energy, scaled down to a 3-micropulse bundle, was applied to the leakage area with a 1-spot-spacing density. Retreatments were performed every 2–3 months using the same laser parameters as used in the first treatment if SRF persisted in the fovea on OCT.

## Results

Among 204 patients with CSC who underwent SRT, six eyes of six patient with CSC with nonconforming FCEs (2.9%) were ultimately included in the data analysis. The mean age of patients with FCEs was 48.5 ± 8.87 years (range: 36–60 years). All six eyes were myopic, with an average spherical equivalent of -2.29 ± 2.21 diopters. Their mean CFT was 243.17 ± 52.83 μm and their mean SFCT was 373.17 ± 36.35 μm at baseline. The MD of retinal sensitivity was − 0.76 ± 0.5 dB at baseline. None of the patients had ever received systemic or topical treatments such as aldosterone receptor antagonists, carbonic anhydrase inhibitors, or topical NSAIDs. Four of the six eyes had previously undergone intravitreal anti-VEGF injections. In all cases, the site of angiographic leakage was found to coincide with the location of the FCE.

Table 1 summarizes the clinical characteristics and pre- and post-treatment changes after SRT in each of the six eyes with nonconforming FCEs. In the eyes of five patients, complete SRF resolution was observed during the follow-up period, while one patient demonstrated minimal remaining SRF. Four patients showed improvements in BCVA; however, the BCVA in the other two patients remained unchanged. The mean number of SRT spots was 35.5 ± 18.64 and the mean pulse energy was 143.33 ± 15.06 µJ for the initial SRT. The treatment spots were applied at leaks in the macular area based on barely visible images of test spots; however, no SRT spots were visible on CFP during the follow-up period. Although abnormally increased autofluorescence in the central fovea noted in Case 2 was observed to decrease after SRT, no decreased autofluorescence indicative of RPE atrophy was observed during follow-up in any of the patients in this study.

**Table 1:** Clinical characteristics and pre- and post-treatment changes following selective retina therapy (SRT) in eyes with nonconforming focal choroidal excavation (FCE) accompanied by central serous chorioretinopathy (CSC)

CASE	Age	Sex	Refractive error (spherical equivalent, D)	Previous treatment	Number of Session of SRT	The number of spots/pulse energy (µJ)at 1st SRT	Maximum Excavation depth/width (µm)	Time to SRF resolution after first SRT (months)	The of SRF existence after final SRT	Follow up period (months)	Baseline				Final follow-up			
											BCVA	CFT (µm)	SFCT (µm)	MD (dB)	BCVA	CFT (µm)	SFCT (µm)	MD (dB)
1	56	F	-1.75	None	5	27/120	88/644	12	Miminally remained	12	20/32	254	374	-0.19	20/25	198	370	+ 0.95
2	36	M	-5.75	5 IVT anti-VEGF	4	25/160	200/810	4	resolved	12	20/63	210	402	-0.93	20/32	110	405	+ 1.91
3	51	M	-1.0	3 IVT anti-VEGF	2	60/140	83/610	6	resolved	6	20/32	206	357	-1.16	20/25	157	351	-0.56
4	60	M	-0.13	1 IVT anti-VEGF	3	30/160	66/420	9	resolved	9	20/40	343	374	-0.43	20/32	196	375	+ 0.31
5	45	F	-0.88	5 IVT anti-VEGF	1	14/140	62/670	3	resolved	6	20/25	206	314	-0.38	20/25	183	316	-0.28
6	43	M	-4.25	None	1	57/140	52/306	1	resolved	6	20/25	240	418	-1.47	20/25	190	415	-0.70

BCVA, best-corrected visual acuity; CFT, central foveal thickness; SFCT, subfoveal choroidal thickness; MD, mean deviation ; IVT anti-VEGF, intravitreal anti-VEGF injection

All patients showed improvements in the MD of retinal sensitivity during the follow-up period. Additionally, no scotomatous points with a decrease of > 6 dB in retinal sensitivity were observed in the SRT-treated eyes in this study.

### Case 1

**Fig. 1:**
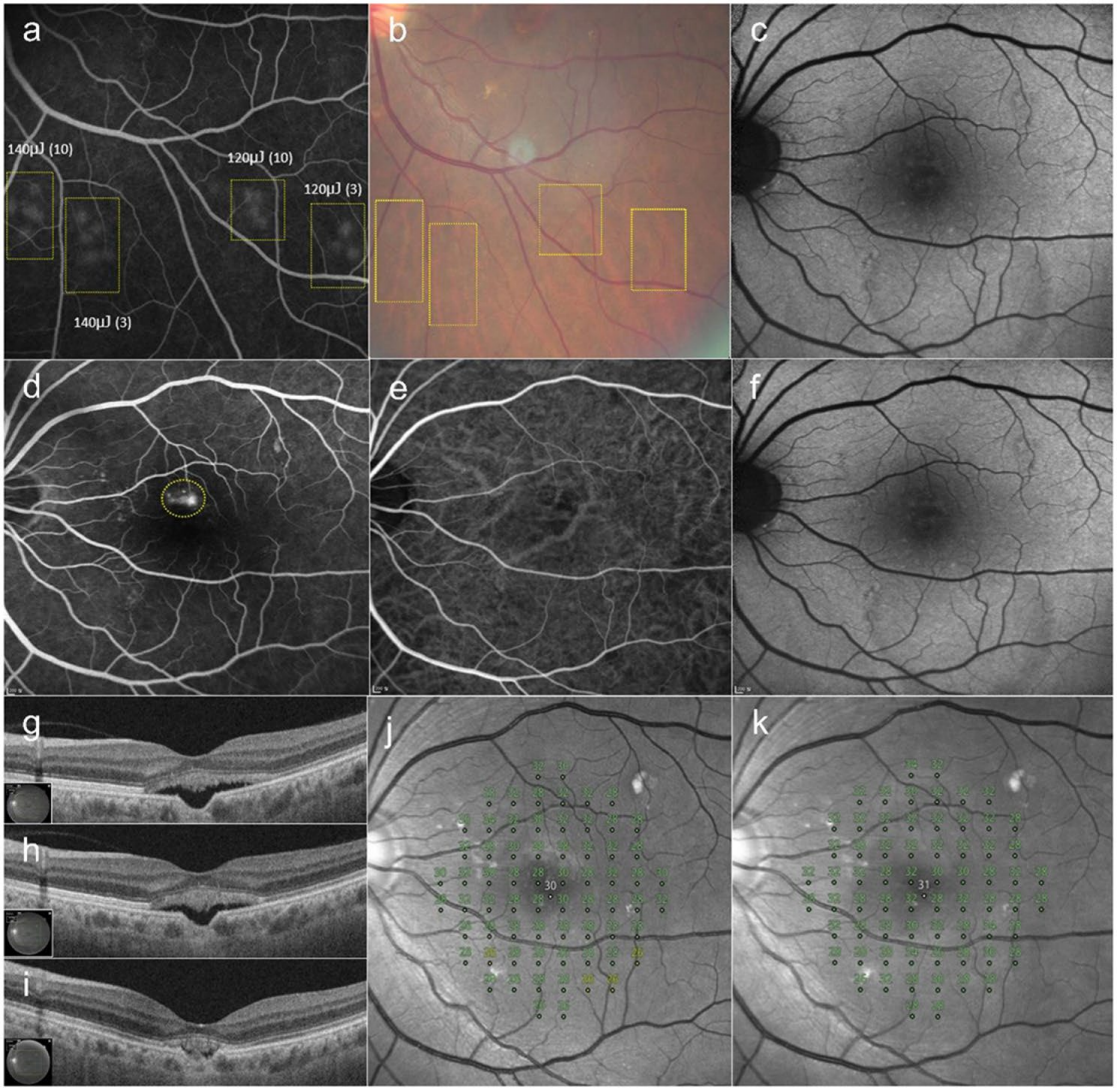
A 56-year-old female patient presented with a 9-month history of metamorphopsia in the left eye. (**a**) Five pairs of selective retina therapy (SRT) test spots of 120 µJ with a bundle of 10 and of 3 micropulses are visible around the inferior arcade vessel on fundus fluorescein angiography (FFA) by 1 h after irradiation (yellow boxes). (**b**) On color fundus photography (CFP), by 1 h after irradiation, test spots of 120 µJ with 10 micropulses are barely visible, and spots of 120 µJ with 3 micropulses are less visible than those generated by using 120 µJ with 10 micropulses. (**c**) Fundus autofluorescence at baseline. (**d**) Late-phase FFA shows diffuse hyperfluorescence around focal choroidal excavation (FCE); 27 treatment spots were applied to the leakage area (yellow circle) using 120 µJ with 3 micropulse bundles. (**e**) Indocyanine green angiography shows dilated choroidal vessels around the FCE. (**f**) No visible change was observed on fundus autofluorescence at 12 months after the1st SRT session. (**g**) SRF and FCE in the fovea are observed on swept-source optical coherence tomography at baseline. (**h**) At 6 months after SRT, the SRF is decreased. (**i**) At 12-months post-SRT, minimal SRF is observed over five SRT sessions performed at two-month intervals. The mean deviation of retinal sensitivity improved from − 0.19 dB at baseline (**j**) to + 0.95 dB at 12 months (**k**) after SRT. No scotomatous points were observed in the SRT-treated area on microperimetry

A 56-year-old female presented with a 9-month history of metamorphopsia in the left eye. SRF and an FCE in the fovea were observed on OCT. Late-phase FFA showed focal and diffuse hyperfluorescence around the FCE. ICGA showed choroidal hyperpermeability. Twenty-seven treatment spots (120 µJ in 3-micropulse bundles) were created in the leakage area. The SRF gradually decreased over five SRT sessions performed at two-month intervals. Minimal SRF was observed at 12-months post-treatment. Her BCVA improved from 20/32 at baseline to 20/25 at 12-months post-SRT. The MD of her retinal sensitivity improved from − 0.19 dB at baseline to + 0.95 dB at 12-months post-SRT (Fig. 1).

### Case 2

**Fig. 2:**
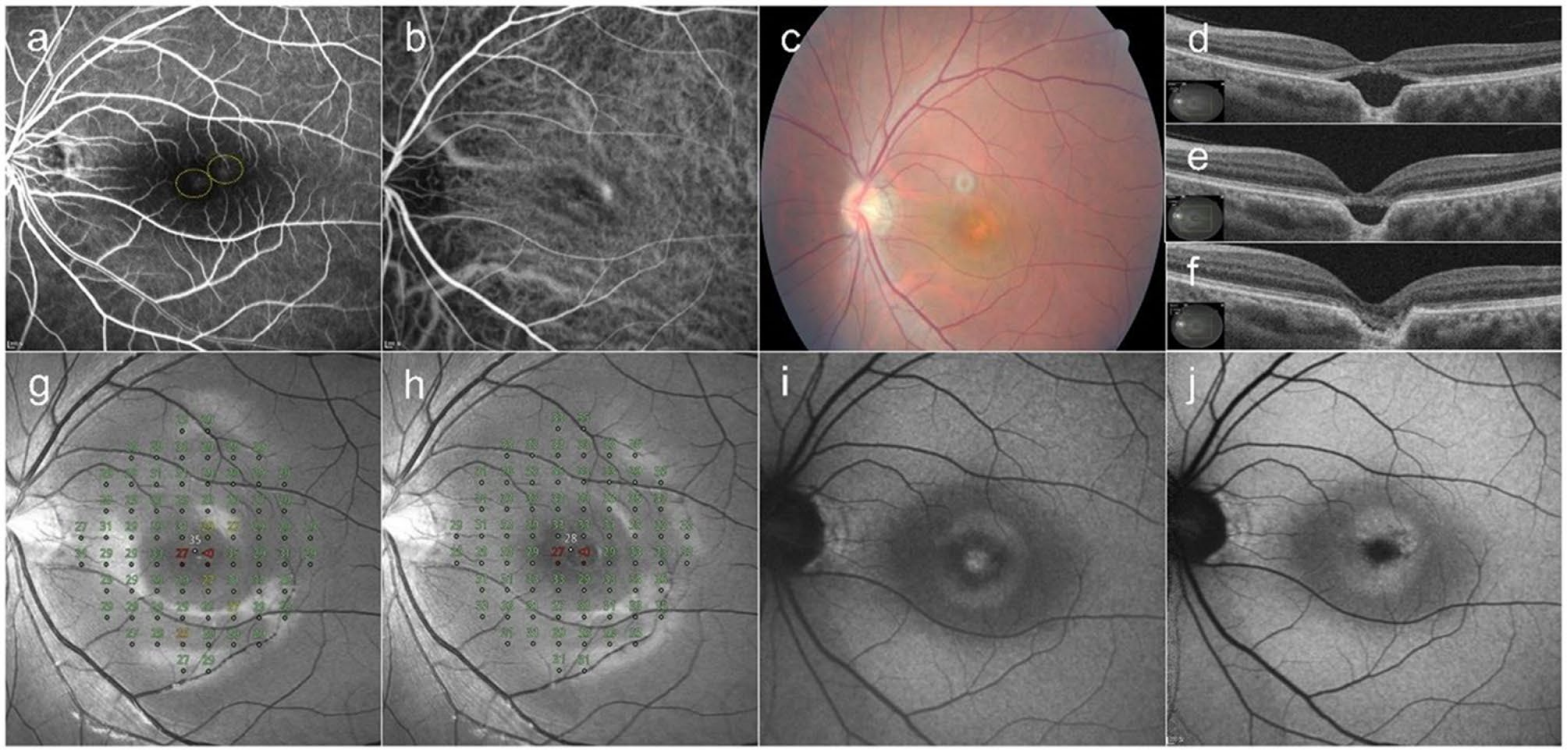
A 36-year-old male patient with a 1-year history of blurred vision in the left eye. (**a**) Late-phase fundus fluorescein angiography showed diffuse hyperfluorescence at the margin of the focal choroidal excavation (FCE). Twenty-five selective retina therapy (SRT) spots were lasered on the leakage area. (two yellow circles). (**b**) Indocyanine green angiography shows choroidal hyperpermeability and dilated choroidal vessels at baseline. (**c**) Color fundus photography (CFP) at baseline. (**d**) Subretinal fluid (SRF) and FCE in the fovea are observed on optical coherence tomography at baseline. (**e**) At 2-months post-SRT, a second SRT session was performed because SRF persisted. (**f**) Complete resolution of SRF was observed at 4-months post-treatment. The mean deviation of retinal sensitivity improved from − 0.93 dB at baseline (**g**) to + 1.91 dB at 12 months (**h**) after SRT. (**i**) Fundus autofluorescence at baseline shows an abnormally increased autofluorescence at the FCE area. (**j**) The autofluorescence in the FCE region is decreased after the SRF had completely resolved at 4-months post-SRT

A 36-year-old male presented with a 1-year history of blurred vision in his left eye. OCT showed SRF and an FCE in the fovea. He had received five anti-VEGF injections at a local clinic 3 months prior to his visit, but the SRF persisted. Late-phase FFA showed diffuse hyperfluorescence at the margin of the FCE. ICGA showed hyperfluorescence and choroidal hyperpermeability. Twenty-five treatment spots of 160 µJ in 3-micropulse bundles were applied to the leakage area in the macula, because barely visible changes were first observed in a test spot created with 160 µJ in 10-micropulse bundles. At 2-months post-SRT, a second SRT procedure was performed because of persistent SRF. Complete resolution of SRF was observed at 4 months after the first SRT. Since recurrence of a small amount of SRF was observed at 6-months post-SRT, two additional SRT procedures were performed. His BCVA improved from 20/63 at baseline to 20/32 at 12-months post-SRT. The MD of retinal sensitivity improved from − 0.93 dB at baseline to + 1.91 dB at 12-months post-SRT. No scotomatous points in retinal sensitivity were observed on microperimetry at 12 months after SRT (Fig. 2).

### Case 3

**Fig. 3:**
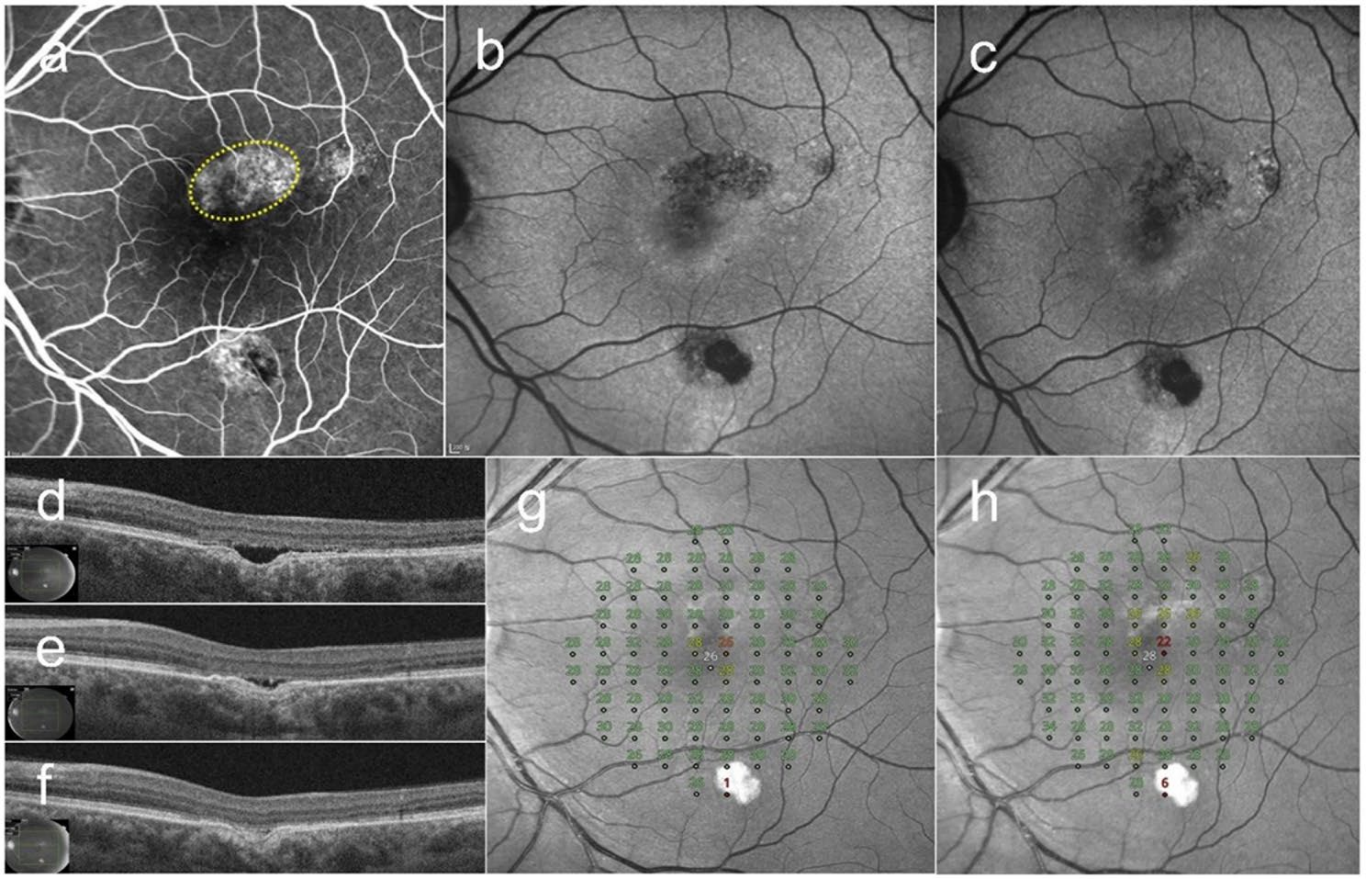
A 51-year-old male patient with an 18-month history of blurred vision in the left eye. (**a**) Late-phase fundus fluorescein angiography shows diffuse hyperfluorescence in the fovea. Sixty selective retina therapy (SRT) treatment spots were irradiated onto the leakage area. (yellow circle). (**b**) Baseline fundus autofluorescence shows hypoautofluorescence corresponding to pigmentary mottling around the FCE area. (**c**) At 6-month post-SRT, hypoautofluorescence becomes more prominent at the site of pigmentary mottling. (**d**) Subretinal fluid (SRF) and focal choroidal excavation in the fovea are observed on optical coherence tomography at baseline. (**e**) At 3-month post-SRT, an additional SRT session was performed due to persistent SRF. (**f**) Complete resolution of SRF is observed at 6 months. The mean deviation of retinal sensitivity increased from − 1.16 dB at baseline (**g**) to -0.56 dB at 6 months (**h**) after SRT

A 51-year-old male presented with an 18-month history of blurred vision in the left eye. He had received three anti-VEGF injections at another clinic years ago, but did not respond to this treatment. OCT revealed SRF and an FCE in the fovea. Late-phase FFA showed diffuse hyperfluorescence in the fovea. FAF revealed hypoautofluorescence around FCE. Sixty SRT spots were delivered to the leakage area. After two sessions of SRT, at 6-months post-treatment, the SRF was completely resolved. His BCVA was improved from 20/32 at baseline to 20/25 at 6-months post-SRT. The MD of retinal sensitivity improved from − 1.16 dB at baseline to − 0.56 dB at 6-months post-SRT (Fig. 3).

### Case 4

**Fig. 4:**
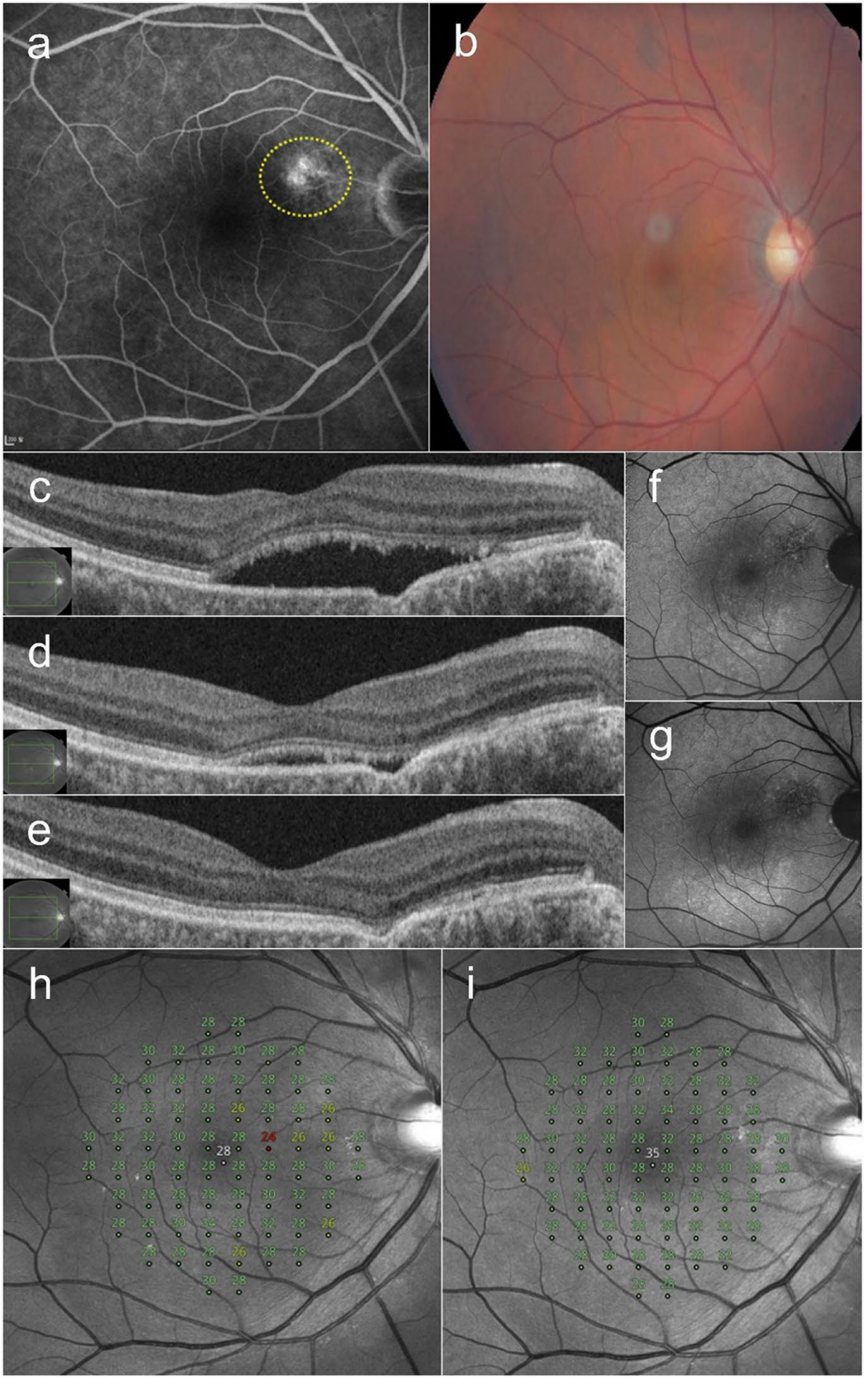
A 60-year-old male patient with a 12-month history of blurred vision in the right eye. (**a**) Late-phase fundus fluorescein angiography shows diffuse hyperfluorescence around the focal choroidal excavation (FCE). Thirty selective retina therapy (SRT) spots were lasered onto the leakage area. (yellow circle). (**b**) Color fundus photography at baseline. (**c**) Optical coherence tomography reveals subretinal fluid (SRF) in the fovea and a nonconforming FCE in the juxtafoveal region at baseline. (**d**) At 6 months after the first SRT, SRF persisted, and a third session of SRT was performed. (**e**) Complete resolution of SRF was observed at 9 months after the initial SRT session. (**f**) Baseline fundus autofluorescence shows hypoautofluorescence corresponding to pigmentary mottling around the FCE area. (**g**) At 9 months, after the resolution of SRF, hypoautofluorescence becomes more prominent at the site of pigmentary mottling. The mean deviation of retinal sensitivity improved from − 0.43 dB at baseline (**h**) to + 0.31 dB at 9 months (**i**) after SRT

A 60-year-old male presented with a 12-month history of blurred vision in the right eye. He had received one anti-VEGF injection at a local clinic 4 months earlier. OCT revealed a nonconforming FCE in the juxtafoveal region. FFA showed diffuse leaks around the FCE. Thirty SRT spots were irradiated in the leakage area. Complete resolution of the SRF was observed 9 months after SRT, administered at 3-month intervals. The BCVA improved from 20/40 at baseline to 20/32, and the MD of retinal sensitivity improved from − 0.43 dB to + 0.31 dB at 9 months post-SRT (Fig. 4).

### Case 5

**Fig. 5:**
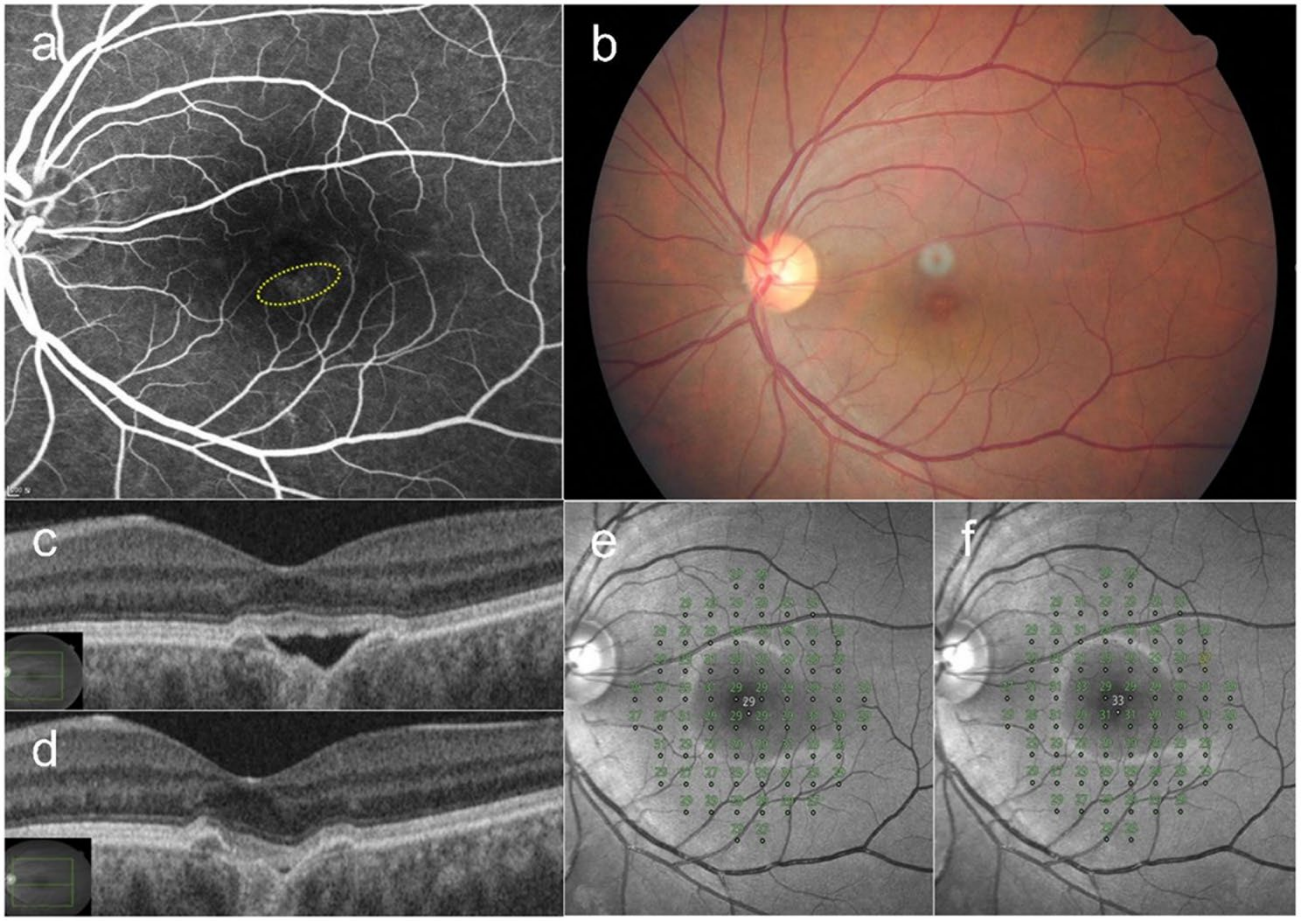
A 45-year-old female patient with a 9-month history of metamorphopsia in the left eye. (**a**) Late-phase fundus fluorescein angiography shows diffuse hyperfluorescence at the lower margin of a focal choroidal excavation (FCE). Fourteen selective retina therapy (SRT) treatment spots were irradiated onto the leakage area (yellow circle). (**b**) Color fundus photography at baseline. (**c**) Optical coherence tomography shows a nonconforming FCE and subretinal fluid (SRF) in the central fovea. (**d**) Complete resolution of SRF is observed at 3 months after SRT. The mean deviation of retinal sensitivity improved from − 0.38 dB at baseline (**e**) to -0.28 dB at 6 months (**f**) after SRT

A 45-year-old female presented with a 9-month history of metamorphopsia in the left eye. She had received five anti-VEGF injections at a local clinic 6 months earlier. OCT revealed a nonconforming FCE. FFA showed diffuse leakages at the lower margin of the FCE. Fourteen SRT spots were created in the leakage area. Since minimal SRF persisted during the follow-up period, no additional SRT session was performed. The SRF was completely resolved at 3-months post-SRT. At 6-months post-treatment, his BCVA was maintained from 20/25 at baseline to 20/25, and the MD of retinal sensitivity was improved from − 0.38 dB at baseline to -0.28 dB (Fig. 5).

### Case 6

**Fig. 6:**
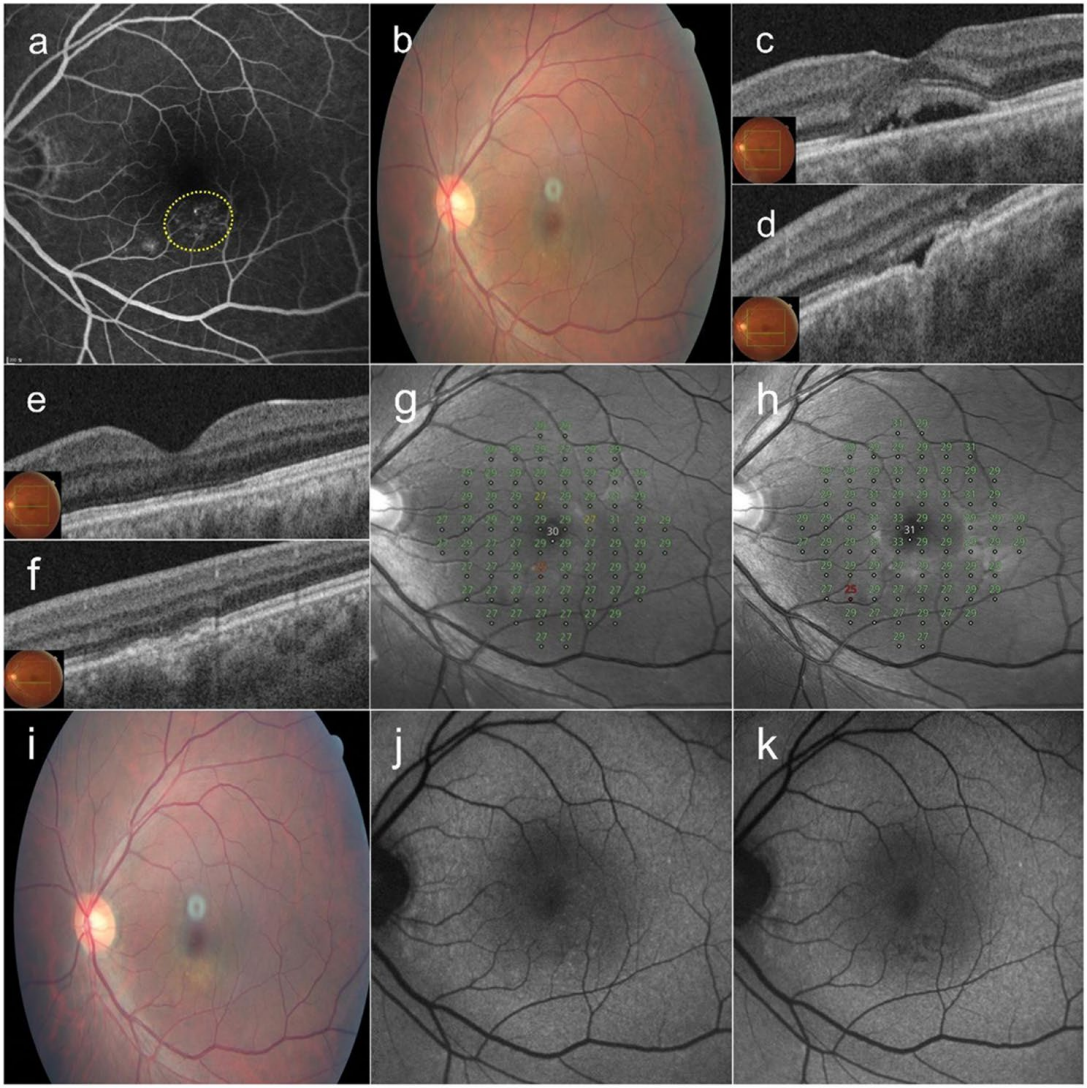
A 43-year-old male patient with a 6-month history of subretinal fluid (SRF) in the left eye. (**a**) Late-phase fundus fluorescein angiography shows diffuse hyperfluorescence around a focal choroidal excavation (FCE) in the juxtafoveal region. Fifty-seven selective retina therapy (SRT) treatment spots were applied to the leakage area (yellow circle). (**b**) Color fundus photography at baseline. (**c**) At baseline, optical coherence tomography shows SRF in the fovea and a nonconforming FCE (**d**) in the juxtafoveal area. (**e**, **f**) Complete resolution of SRF is observed at 1 month after SRT. The mean deviation of retinal sensitivity improved from − 1.47 dB at baseline (**g**) to -0.70 dB at 12 months (**h**) after SRT. (**i**) The fundus photograph at 12 months showed no change in the FCE region. (**j**) Baseline fundus autofluorescence shows hypoautofluorescence corresponding to pigmentary mottling around the FCE area. (**k**) At 12 months, after SRF resolution, hypoautofluorescence becomes more prominent at the site of pigmentary mottling

A 43-year-old male patient with no history of prior treatment presented with a 6-month history of SRF in the left eye. OCT revealed SRF in the fovea and a nonconforming FCE in the juxtafoveal area. FFA showed diffuse leakages around the FCE. Fifty-seven SRT spots were delivered in the leakage area. The SRF resolved completely by 1 month after SRT. His BCVA was 20/25 at both baseline and 12 months after SRT, while the MD of retinal sensitivity improved from − 1.47 dB to − 0.70 dB over the same period (Fig. 6).

## Discussion

In our study, the prevalence of nonconforming FCEs in patients with CSC was 2.9%, which is slightly higher than the prevalence of 2.8% reported in a prior study of 248 eyes [8]. Although few papers have addressed CSC with FCE, patients with CSC with FCEs have been predominantly described as having myopia [23–25]. All six patients in our study had myopia. While one patient showed minimal persistent SRF, the SRF in the other five patients resolved completely during the follow-up period. Thus, SRT monotherapy showed favorable outcomes in CSC with nonconforming FCEs.

Although myopia is generally associated with a thinner choroid, our cases nevertheless exhibited pachychoroid features in association with FCE. This unexpected finding has also been documented in prior report [25], which described that pachychoroid characteristics and FCE may occur even in myopic eyes. These findings support that pachychoroid pathophysiology can coexist with myopic refractive status in CSC with FCE.

Although several studies have reported that anti-VEGF is effective in the treatment of FCE complicated by polypoidal choroidal vasculopathy or CNV [26, 27], few reports on the treatment of CSC with nonconforming FCE are available. Liu et al. reported in a cohort study of FCE that SRF resolved in a single CSC patient with concomitant FCE following subthreshold micropulse laser (SMPL) treatment [28]. Unlike SRT, SMPL exerts its effect without producing detectable RPE damage. It is thought to act primarily by stimulating RPE cells and promoting the release of heat shock proteins. SMPL is typically applied using a fixed duty cycle of 5–15%, with the treatment power titrated to approximately 30–50% of the threshold for a barely visible spot [11]. While SMPL has been shown to be effective in resolving SRF in CSC, its potential role in CSC with concomitant FCE remains to be further elucidated. Suzuki et al. [8] reported that, of seven patients with CSC with FCE, two patients showed SRF resolution after PDT treatment, while the other five patients revealed spontaneous SRF resolution over different follow-up periods, ranging from 1 month to 37 months. However, their study did not include functional outcomes, such as visual acuity and visual field.

In the present study, four patients demonstrated improved BCVA, while the other two patients maintained their BCVA. Since the absence of scotomatous changes in microperimetry testing and the increase in mean MD values of retinal sensitivity after SRT sessions were observed in all patients, our results indicated that SRT did not cause photoreceptor damage.

Margolis et al. reported that five of six eyes with FCE demonstrated hyperfluorescence consistent with transmission defects due to RPE attenuation [7]. This indicated that FCE might be associated with RPE abnormalities. Choroidal thinning was also observed in CSC eyes with FCE in previous studies [23, 29]; however, our patients had thick choroids, with the SFCT ranging from 314 μm to 418 μm. Since the cause of CSC is known to be related to RPE dysfunction and choroidal hyperpermeability, CSC with FCEs seems to be caused by RPE and/or choroidal abnormalities. Regarding the mechanism by which SRT removes SRF from nonconforming FCEs, we speculate that selective RPE damage from SRT induces migration and proliferation of RPE cells adjacent to the SRT-damaged area. Then, the newly formed RPE layer could restore the outer blood–retinal barrier, preventing RPE leakage and eliminating SRF [17]. In the current study, since no dark autofluorescence indicative of RPE atrophy was observed on FAF after SRT, RPE restoration appears to have been well-achieved in the SRT-treated area.

CSC, known as pachychoroid disease, showed a pathologically thickened choroid [30, 31]. The effect of SRT on the choroid has not been well-studied to date. Similar to previous reports of the absence of significant changes in the SFCT after SRT [32, 33], the change from a mean SFCT of 373.17 ± 36.35 μm at baseline to a mean SFCT of 372.0 ± 36.13 μm at the last visit was 1.17 μm in this study. This very small change in SFCT indicates that SRT might exerts its effects mainly by changing the RPE layer, rather than the choroid.

Previously, we reported that the multiple sessions of SRT was beneficial in patients with CSC [34]. In this study, SRF was resolved in two patients after a single SRT session, but four patients required additional SRT. One patient (Case 2) showed recurrence; however the SRF resolved after additional SRT. Therefore, multiple sessions of SRT were helpful to remove SRF in cases of CSC with FCE.

Our study had some limitations, including its retrospective design, short follow-up, and small number of cases. Four of six patients had received intravitreal anti-VEGF injections more than 3 months before SRT, all of whom achieved complete SRF resolution, while among the two treatment-naïve patients, one showed complete resolution and one had minimal residual SRF. Of note, the anti-VEGF previously administered in these four cases was bevacizumab, but given the interval of more than 3 months before SRT, its influence on the outcomes may have been limited. However, considering the rarity of CSC with FCE and the difficulty in collecting sufficient clinical data, our results are valuable in suggesting SRT as a potential treatment option.

## Conclusion

In this case series study, SRT monotherapy was shown to be useful for removing SRF in patients with CSC with nonconforming FCE.

## Data Availability

No datasets were generated or analysed during the current study.

## References

[1] Wang M, Munch IC, Hasler PW, Prünte C, Larsen M. Central serous chorioretinopathy. Acta Ophthalmol. 2008;86(2):126–45. 17662099 10.1111/j.1600-0420.2007.00889.x

[2] Kitzmann AS, Pulido JS, Diehl NN, Hodge DO, Burke JP. The incidence of central serous chorioretinopathy in olmsted County, Minnesota, 1980–2002. Ophthalmology. 2008;115(1):169–73. 18166410 10.1016/j.ophtha.2007.02.032

[3] Liu B, Deng T, Zhang J, RISK FACTORS FOR CENTRAL. SEROUS CHORIORETINOPATHY: A systematic review and Meta-Analysis. Retina. 2016;36(1):9–19. 26710181 10.1097/IAE.0000000000000837

[4] Schellevis RL, Altay L, Kalisingh A, Mulders TWF, Sitnilska V, Hoyng CB, et al. Elevated steroid hormone levels in active chronic central serous chorioretinopathy. Invest Ophthalmol Vis Sci. 2019;60(10):3407–13. 31387112 10.1167/iovs.19-26781

[5] Jampol LM, Shankle J, Schroeder R, Tornambe P, Spaide RF, Hee MR. Diagnostic and therapeutic challenges. Retina. 2006;26(9):1072–6. 17151497 10.1097/01.iae.0000248819.86737.a5

[6] Katome T, Mitamura Y, Hotta F, Niki M, Naito T. Two cases of focal choroidal excavation detected by spectral-domain optical coherence tomography. Case Rep Ophthalmol. 2012;3(1):96–103. 23008695 10.1159/000337880 PMC3448114

[7] Margolis R, Mukkamala SK, Jampol LM, Spaide RF, Ober MD, Sorenson JA, et al. The expanded spectrum of focal choroidal excavation. Arch Ophthalmol. 2011;129(10):1320–5. 21670327 10.1001/archophthalmol.2011.148

[8] Suzuki M, Gomi F, Hara C, Sawa M, Nishida K. Characteristics of central serous chorioretinopathy complicated by focal choroidal excavation. Retina. 2014;34(6):1216–22. 24240563 10.1097/IAE.0000000000000045

[9] Klein ML, Van Buskirk EM, Friedman E, Gragoudas E, Chandra S. Experience with nontreatment of central serous choroidopathy. Arch Ophthalmol. 1974;91(4):247–50. 4621147 10.1001/archopht.1974.03900060257001

[10] Gilbert CM, Owens SL, Smith PD, Fine SL. Long-term follow-up of central serous chorioretinopathy. Br J Ophthalmol. 1984;68(11):815–20. 6541945 10.1136/bjo.68.11.815 PMC1040477

[11] Feenstra HMA, van Dijk EHC, Cheung CMG, Ohno-Matsui K, Lai TYY, Koizumi H, et al. Central serous chorioretinopathy: an evidence-based treatment guideline. Prog Retin Eye Res. 2024;101:101236. 38301969 10.1016/j.preteyeres.2024.101236

[12] Ficker L, Vafidis G, While A, Leaver P. Long-term follow-up of a prospective trial of argon laser photocoagulation in the treatment of central serous retinopathy. Br J Ophthalmol. 1988;72(11):829–34. 3061449 10.1136/bjo.72.11.829 PMC1041600

[13] Khosla PK, Rana SS, Tewari HK, Azad RU, Talwar D. Evaluation of visual function following argon laser photocoagulation in central serous retinopathy. Ophthalmic Surg Lasers. 1997;28(8):693–7. 9269005

[14] Ruiz-Moreno JM, Lugo FL, Armadá F, Silva R, Montero JA, Arevalo JF, et al. Photodynamic therapy for chronic central serous chorioretinopathy. Acta Ophthalmol. 2010;88(3):371–6. 19958296 10.1111/j.1755-3768.2008.01408.x

[15] Reibaldi M, Cardascia N, Longo A, Furino C, Avitabile T, Faro S, et al. Standard-fluence versus low-fluence photodynamic therapy in chronic central serous chorioretinopathy: a nonrandomized clinical trial. Am J Ophthalmol. 2010;149(2):307–. – 15.e2. 19896635 10.1016/j.ajo.2009.08.026

[16] Lim JI, Glassman AR, Aiello LP, Chakravarthy U, Flaxel CJ, Spaide RF. Collaborative retrospective macula society study of photodynamic therapy for chronic central serous chorioretinopathy. Ophthalmology. 2014;121(5):1073–8. 24439758 10.1016/j.ophtha.2013.11.040

[17] Brinkmann R, Roider J, Birngruber R. Selective retina therapy (SRT): a review on methods, techniques, preclinical and first clinical results. Bull Soc Belge Ophtalmol. 2006;302:51–69. 17265790

[18] Jeon SH, Kim M, Roh YJ. Retinal pigment epithelial responses based on the irradiation density of selective retina therapy. Graefes Arch Clin Exp Ophthalmol. 2021;259(1):101–11. 32794108 10.1007/s00417-020-04887-2

[19] Klatt C, Saeger M, Oppermann T, Pörksen E, Treumer F, Hillenkamp J, et al. Selective retina therapy for acute central serous chorioretinopathy. Br J Ophthalmol. 2011;95(1):83–8. 20554506 10.1136/bjo.2009.178327

[20] Yasui A, Yamamoto M, Hirayama K, Shiraki K, Theisen-Kunde D, Brinkmann R, et al. Retinal sensitivity after selective retina therapy (SRT) on patients with central serous chorioretinopathy. Graefes Arch Clin Exp Ophthalmol. 2017;255(2):243–54. 27497611 10.1007/s00417-016-3441-8

[21] Nam SW, Lee JH, Byun Z, Ham DI, Kong M. Evaluation of the microperimetry in eyes with cuticular Drusen. Sci Rep. 2022;12(1):17557. 36266529 10.1038/s41598-022-22513-5 PMC9584893

[22] Jeon SH, Kim M, Roh YJ. Use of a fundus Image-Based Titration strategy for selective retina therapy for central serous chorioretinopathy. J Clin Med. 2024;13:17. 10.3390/jcm13175230 PMC11396731 39274443

[23] Ellabban AA, Tsujikawa A, Ooto S, Yamashiro K, Oishi A, Nakata I, et al. Focal choroidal excavation in eyes with central serous chorioretinopathy. Am J Ophthalmol. 2013;156(4):673–83. 23831223 10.1016/j.ajo.2013.05.010

[24] Wakabayashi Y, Nishimura A, Higashide T, Ijiri S, Sugiyama K. Unilateral choroidal excavation in the macula detected by spectral-domain optical coherence tomography. Acta Ophthalmol. 2010;88(3):e87–91. 20546234 10.1111/j.1755-3768.2010.01895.x

[25] Chung H, Byeon SH, Freund KB, FOCAL CHOROIDAL EXCAVATION AND ITS ASSOCIATION WITH PACHYCHOROID. SPECTRUM DISORDERS: A review of the literature and multimodal imaging findings. Retina. 2017;37(2):199–221. 27749784 10.1097/IAE.0000000000001345

[26] Kobayashi W, Abe T, Tamai H, Nakazawa T. Choroidal excavation with polypoidal choroidal vasculopathy: a case report. Clin Ophthalmol. 2012;6:1373–6. 22969281 10.2147/OPTH.S33879 PMC3437952

[27] Xu H, Zeng F, Shi D, Sun X, Chen X, Bai Y. Focal choroidal excavation complicated by choroidal neovascularization. Ophthalmology. 2014;121(1):246–50. 24095605 10.1016/j.ophtha.2013.08.014

[28] Liu P, An G, Lu C, Li S, Chen H, Jin B, et al. Analysis of clinical features and SS-OCT findings in patients with focal choroidal excavation. Sci Rep. 2025;15(1):12187. 40204830 10.1038/s41598-025-95561-2 PMC11982228

[29] Luk FO, Fok AC, Lee A, Liu AT, Lai TY. Focal choroidal excavation in patients with central serous chorioretinopathy. Eye (Lond). 2015;29(4):453–9. 25853402 10.1038/eye.2015.31 PMC4816359

[30] Imamura Y, Fujiwara T, Margolis R, Spaide RF. Enhanced depth imaging optical coherence tomography of the choroid in central serous chorioretinopathy. Retina. 2009;29(10):1469–73. 19898183 10.1097/IAE.0b013e3181be0a83

[31] Maruko I, Iida T, Sugano Y, Ojima A, Ogasawara M, Spaide RF. Subfoveal choroidal thickness after treatment of central serous chorioretinopathy. Ophthalmology. 2010;117(9):1792–9. 20472289 10.1016/j.ophtha.2010.01.023

[32] Park YG, Kang S, Kim M, Yoo N, Roh YJ. Selective retina therapy with automatic real-time feedback-controlled dosimetry for chronic central serous chorioretinopathy in Korean patients. Graefes Arch Clin Exp Ophthalmol. 2017;255(7):1375–83. 28421342 10.1007/s00417-017-3672-3

[33] Hirayama K, Yamamoto M, Kohno T, Kyo A, Theisen-Kunde D, Brinkmann R, et al. Selective retina therapy (SRT) for macular serous retinal detachment associated with Tilted disc syndrome. Graefes Arch Clin Exp Ophthalmol. 2021;259(2):387–93. 32960320 10.1007/s00417-020-04931-1

[34] Jeon SH, Kim M, Lee J, Roh YJ. The effect of selective retina therapy for Bevacizumab-Resistant chronic central serous chorioretinopathy. Ophthalmologica. 2022;245(1):91–100. 34649253 10.1159/000520187

